# Supplementary material for: Regulatory Standard for Determining Preoperative Skin Preparation Efficacy Underreports True Dermal Bioburden in a Porcine Model
Source: Microorganisms. 2024 Nov 20;12(11):2369. doi: 10.3390/microorganisms12112369 (PMC11596398; doi:10.3390/microorganisms12112369)
Supplement: Supplementary file 1 [file microorganisms-12-02369-s001.zip › microorganisms-3294163-supplementary.pdf]

Supplementary Material

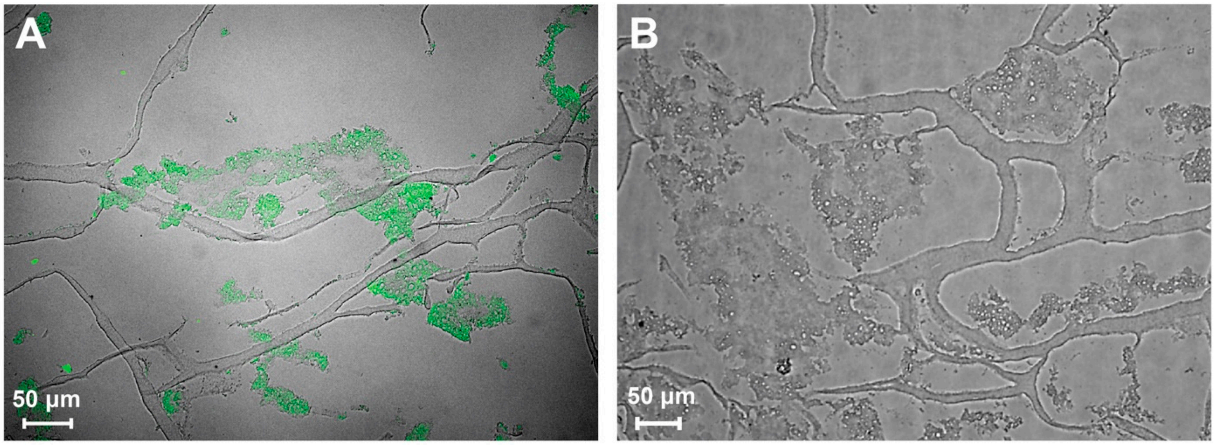

Suplementary Figure S1. Cross-sections of *Staphylococcus epidermidis* grown on a collagen plug were stained as positive and negative process controls. (A) Underwent the normal IF treatment protocol including incubation with a *S. epidermidis* monoclonal antibody (primary) followed by a Goat Anti-Mouse IgG Alexa Fluor 488 polyclonal antibody (secondary). Fluorescence was observed. (B) Underwent a modified IF treatment including incubation with the Goat Anti-Mouse IgG Alexa Fluor 488 Polyclonal secondary antibody only. No fluorescence was observed.
